# Exercise worsening of electromechanical disturbances: A predictor of arrhythmia in long QT syndrome

**DOI:** 10.1002/clc.23132

**Published:** 2018-12-22

**Authors:** Dafni Charisopoulou, George Koulaouzidis, Annika Rydberg, Henein Y. Michael

**Affiliations:** ^1^ Institute of Public Health and Clinical Medicine Umea University Umea Sweden; ^2^ Department of Paediatric Cardiology Leeds Teaching Hospitals NHS Trust Leeds UK; ^3^ Department of Cardiology Mid Yorkshire Hospitals NHS Trust Wakefield, UK; ^4^ Department of Clinical Sciences Paediatrics, Umea University Umea Sweden; ^5^ Molecular and Clinical Sciences Research Institute St George University London London UK; ^6^ Brunel University Middlesex UK

**Keywords:** arrhythmia, electromechanical window, exercise echocardiography, long QT syndrome

## Abstract

**Background:**

Electromechanical (EM) coupling heterogeneity is significant in long QT syndrome (LQTS), particularly in symptomatic patients; EM window (EMW) has been proposed as an indicator of interaction and a better predictor of arrhythmia than QTc.

**Hypothesis:**

To investigate the dynamic response of EMW to exercise in LQTS and its predictive value of arrhythmia.

**Methods:**

Forty‐seven LQTS carriers (45 ± 15 years, 20 with arrhythmic events), and 35 controls underwent exercise echocardiogram. EMW was measured as the time difference between aortic valve closure on Doppler and the end of QT interval on the superimposed electrocardiogram (ECG). Measurements were obtained at rest, peak exercise (PE) and 4 minutes into recovery.

**Results:**

Patients did not differ in age, gender, heart rate, or left ventricular ejection fraction but had a negative resting EMW compared with controls (−42 ± 22 vs 17 ± 5 ms, *P* < 0.0001). EMW became more negative at PE (−89 ± 43 vs 16 ± 7 ms, *P* = 0.0001) and recovery (−65 ± 39 vs 16 ± 6 ms, *P* = 0.001) in patients, particularly the symptomatic, but remained unchanged in controls. PE EMW was a stronger predictor of arrhythmic events than QTc (AUC:0.765 vs 0.569, *P* < 0.001). B‐blockers did not affect EMW at rest but was less negative at PE (BB: −66 ± 21 vs no‐BB: −113 ± 25 ms, *P* < 0.001). LQT1 patients had worse PE EMW negativity than LQT2.

**Conclusion:**

LQTS patients have significantly negative EMW, which worsens with exercise. These changes are more pronounced in patients with documented arrhythmic events and decrease with B‐blocker therapy. Thus, EMW assessment during exercise may help improve risk stratification and management of LQTS patients.

## 1 INTRODUCTION

Ventricular tachyarrhythmias, syncope, and even sudden death are of concern in inherited long QT syndrome.^1^, ^2^ Balancing between potential risks, side effects of aggressive management, and life style changes remains a challenge.^3^ LQTS mutations‐related cardiac ion channels defects result in prolonged action potential and increased spatiotemporal dispersion of myocardial repolarization, which predispose to arrhythmia and adverse cardiac events.^4^, ^5^ Identifying patients at risk of arrhythmia is often difficult, particularly among those without previous symptoms and with normal or borderline QTc.^6^, ^7^ Moreover, efforts to optimize individual risk stratification using only electrocardiogram (ECG) parameters of heterogeneity have given conflicting results,^7^ thus highlighting the importance of associated mechanical left ventricular (LV) dysfunction.^8^, ^9^, ^10^, ^11^, ^12^, ^13^, ^14^, ^15^, ^16^, ^17^ Electromechanical (EM) coupling heterogeneity has also been shown in health but appears significantly more pronounced in LQTS.^14^, ^15^, ^16^, ^17^ Noninvasive cardiac EM window (EMW) has been proposed as an indicator of such EM coupling disturbances.^16^, ^17^

EMW corresponds to the time difference between the end of electrical systole (QT interval) and the completion of mechanical systole (onset of aortic valve closure), which is positive in healthy individuals.^17^ Significantly negative EMW has been shown to precede ventricular tachyarrhythmias in drug‐induced LQT.^18^, ^19^ Similar findings have been shown in genotype‐positive LQTS patients, particularly those with arrhythmia.^17^, ^19^ Finally, sympathetic stimulation has been shown to provoke arrhythmia in LQTS^21^ and to worsen the negativity of EMW.^22^, ^23^ We, therefore, aimed to assess the dynamic response of EMW to exercise in LQTS in general and according to its genotype (LQT1 or LQT2), in an attempt to identify carriers at risk of major arrhythmic events.

## 2 METHODS

### 2.1 Study population

Both patients and controls were followed up at the cardiology department of Umeå University Hospital. Molecular analyses of LQTS genotype were performed at the Umeå Department of Clinical Genetics following the current clinical practices for molecular genetic diagnostics.^24^ Individuals with coronary heart disease and those at high risk for atherosclerosis were excluded. Patients were divided into symptomatic and asymptomatic based on documented history of cardiac events (syncope, cardiac arrest, ventricular tachyarrhythmia), according to the patients' hospital clinical notes. Ongoing therapy with B‐blockers (BB) was recorded. ECG and echocardiography parameters were obtained and analyzed by two independent investigators blinded to genotype and clinical details.

The study protocol complied with the ethical guidelines of the 1975 Declaration of Helsinki and was approved by the Regional Ethical Review Board (Umeå University). All participating subjects had given informed consent to take part.

### 2.2 Exercise echocardiography protocol

All participants underwent a semi‐supine (slightly left lateral tilt) bicycle exercise echocardiography using General Electric–GE ergometer (model 900, Ergoline GmbH, Bitz, Germany) with an increasing workload of 10 W every 2 minutes. Measurements were made at: (a) rest, prior to the exercise, (b) peak exercise (PE), achieving 85% of the maximum predicted heart rate for age, and (c) 4 minutes into recovery.

### 2.3 Electrocardiogram

A 12 lead ECG was continuously monitored throughout exercise, recorded at 25 mm/sec with standard lead positioning using a conventional system. R‐R and QT intervals at each of the three stages were digitally measured, with the QT interval from the onset of the QRS to the point of intersection of the descending limb of T wave with the isoelectric line. QT values were corrected for heart rate using the Bazett formula [QTc = QT/(RR)^1/2^].

### 2.4 Echocardiography

The echocardiographic examination was performed in the semi‐supine position using a Vivid 7 echocardiograph (GE, Horten, Norway) equipped with an adult 1.5‐4.3 MHz phased array transducer. We acquired images as consecutive loops from the standard apical four‐chamber and parasternal long‐ and short‐axis views at the end of each exercise stage. All recordings were made with a superimposed ECG (Lead II). Left ventricular ejection fraction (LV EF) was estimated using Simpson's biplane method.^25^ Aortic valve velocity was obtained using pulsed wave Doppler technique from the apical five‐chamber view with the sample volume placed at the aortic valve level.^26^ The aortic valve closure time (QAoC) was measured with respect to the onset of QRS complex. The EMW was calculated by subtracting the QT interval from the QAoC (12, Supporting Information Figure SS1). Offline analyses were made using a commercially available software system (EchoPAC, version 8.0.1; GE, Waukesha, Wisconsin).

### 2.5 Statistical analysis

For the statistical analysis, we used the Statistical Package of Social Science (SPSS) for windows (version 13.0; SPSS Inc, Chicago, Illinois). We expressed continuous variables as mean ± SD and categorical variables as absolute number and percentage (%). Groups were compared with Student *t* test for normally distributed variables and with Mann‐Whitney *U*‐test if variables were not normally distributed. One‐way analysis of variance was used for multiple comparisons. Pearson's test was used to test correlations. The sensitivity and specificity of QT, QTc, and EMW for predicting previous cardiac events in LQTS carriers were investigated by the receiver operating characteristic (ROC) analysis. The alpha reliability coefficient for 20 randomly selected patients at three exercise phases was also estimated. *P*‐values of <0.05 were considered statistically significant.

### 2.6 EMW measurements reproducibility

A good inter‐observer agreement was found for EMW measurements at 0.97 and intraobserver agreement was 0.98.

## 3 RESULTS

### 3.1 Population characteristics

The study population included 47 LQTS mutation carriers (36 LQT1 and 11 LQT2) who were compared with 35 healthy controls matched for age (45 ± 15 vs 47 ± 13 years, *P* = 0.2) and gender (53 vs 54% females, *P* = 0.3). LQTS patients and controls had normal LV EF (65 ± 6 vs 67 ± 7%, *P* = 0.3). Twenty LQTS patients were classified as symptomatic based on documented history of syncope, cardiac arrest or arrhythmia; three of them had received ICD. 14/20 symptomatic and 11/27 asymptomatic patients were on B‐blocker therapy at the time of the study.

### 3.2 Response to exercise

#### 3.2.1 QT, QTc, and QAoC intervals

Patients had significantly longer QT, QTc, and QAoC intervals than controls, at rest, PE and recovery phase (*P* < 0.01 for all, Table 1). The QTc interval lengthened at PE in patients but shortened in controls (Δ +10 ± 9 vs −5.5 ± 3.8%, *P* < 0.0001). It also remained significantly longer at recovery with respect to baseline in patients but reached baseline values in controls (Δ +6.2 ± 5 vs 0.007 ± 2%, *P* < 0.0001).

**Table 1:** Comparison between LQT syndrome mutation carriers and healthy individuals at rest, peak exercise and 4 min into recovery

	Rest			Peak			Recovery		
parameter	LQTS (*n* = 47)	Control (*n* = 35)	*P*‐value	LQTS (*n* = 47)	Control(*n* = 35)	*P*‐value	LQTS (*n* = 47)	Control (*n* = 35)	*P*‐value
HR	69 ± 10	68 ± 10	0.84	121 ± 17	120 ± 15	0.74	69 ± 10	68 ± 9	0.72
R‐R (ms)	887 ± 160	888 ± 135	0.81	508 ± 80	512 ± 69	0.77	884 ± 127	893 ± 129	0.73
QT (ms)	431 ± 45	348 ± 27	0.0001	387 ± 18	273 ± 25	0.0001	456 ± 42	349 ± 23	0.0001
QTc	453 ± 42	413 ± 17	0.0001	499 ± 45	390 ± 19	0.0001	479 ± 35	414 ± 20	0.0001
QAoC	389 ± 46	365 ± 24	0.01	309 ± 18	288 ± 22	0.0001	392 ± 36	364 ± 22	0.0001
EMW	−42 ± 22	17 ± 5	0.0001	−89 ± 43	16 ± 7	0.0001	−65 ± 39	16 ± 6	0.001

Abbreviations: HR, heart rate; R‐R, R‐R interval on corresponding ECG; QTc, corrected QT interval by Bazett formula; QAoC, time interval from R onset to aortic valve closure midline; EMW, electromechanical window (QAoC‐QT); LQTS, long QT syndrome.

#### 3.2.2 Electromechanical window

The EMW was negative in patients at all three phases in contrast to controls in whom it was and remained positive (*P* = 0.0001, Table 1 and Figure SS2) throughout exercise and recovery. It became more negative at PE in patients but did not change in controls (Δ −45 ± 34 vs 1.1 ± 8%, *P* < 0.001). Patient's EMW was more negative at recovery than baseline, but again remained unchanged in controls (Δ −23 ± 44 vs 0.9 ± 8%, *P* = 0.005).

### 3.3 EMW and cardiac events

There were no differences between symptomatic and asymptomatic patients in age, gender or genotype. QT and QTc intervals were longer in symptomatic compared with asymptomatic patients at rest, PE and during recovery (*P* ≤ 0.03 for both, Table 2). QAoC interval was also longer in symptomatic patients at PE and at recovery (*P* ≤ 0.03 for both, Table 2). The EMW was more negative in symptomatic patients at rest and worsened further at PE and recovery (*P* ≤ 0.02 for all phases, Table 3 and Figure SS3).

**Table 2:** Comparison between symptomatic and asymptomatic LQTS patients at rest, peak exercise and 4 min in recovery

	Rest			Peak			Recovery		
parameter	Symptomatic (*n* = 20)	Asymptomatic (*n* = 27)	*P*‐value	Symptomatic (*n* = 20)	Asymptomatic (*n* = 27)	*P*‐value	Symptomatic (*n* = 20)	Asymptomatic (*n* = 27)	*P*‐value
HR (beats/min)	65 ± 8	70 ± 14	0.1	121 ± 18	128 ± 20	0.2	76 ± 12	80 ± 16	0.3
QT (msec)	453 ± 48	415 ± 58	0.03	413 ± 26	368 ± 21	0.0001	489 ± 26	432 ± 36	0.0001
QTc	479 ± 43	447 ± 36	0.02	504 ± 41	479 ± 14	0.003	495 ± 39	469 ± 16	0.002
QAoC (msec)	400 ± 58	381 ± 35	0.1	314 ± 24	303 ± 9	0.03	410 ± 34	378 ± 32	0.002
EMW (msec)	−54 ± 19	−34 ± 20	0.02	−107 ± 34	−74 ± 25	0.0001	−82 ± 44	−55 ± 38	0.02

Abbreviations: HR, heart rate; QTc, corrected QT interval by Bazett formula; QAoC, time interval from R onset to aortic valve closure midline; EMW, electromechanical window (QAoC‐QT); LQTS, long QT syndrome.

**Table 3:** ROC curve analysis of QT, QTc, and EMW for previous cardiac events in the three exercise phases

Variable	AUC	95% CI
At rest		
QTc rest	0.517	0.346‐0.687
EMW rest	0.757	0.615‐0.900
EMW rest + QTc rest	0.772	0.639‐0.905
At peak exercise		
QTc peak	0.569	0.402‐0.735
EMW peak	0.765	0.620‐0.910
EMW peak + QTc peak	0.767	0.618‐0.908
At recovery		
QTc recovery	0.407	0.241‐0.574
EMW recovery	0.748	0.603‐0.893
EMW rec + QTc rec	0.754	0.609‐0.898

Abbreviations: AUC, area under the curve, CI, confidence interval; QTc, corrected QT interval by Bazett formula; ROC, receiver operating characteristic; EMW, electromechanical window; rec, recovery.

### 3.4 Relationship between QTc and EMW

EMW correlated with QTc (*r* = −0.63, *P* < 0.0001). Symptomatic and asymptomatic patients had a more negative EMW for the same QTc value than controls. Symptomatic patients had more negative slope (*P* = 0.04, Figure SS4).

### 3.5 EMW and high‐risk patients

On the ROC analysis, EMW was stronger than QTc in discriminating symptomatic from asymptomatic patients at all three exercise phases (Table 3). Resting EMW < −56 ms was 78% sensitive and 55% specific in identifying patients with previous cardiac events (AUC, area under the curve: 0.757). At PE, EMW < −94 ms was prevalent in high‐risk patients with sensitivity of 75% and specificity of 70% (AUC: 0.765). Respective values at recovery were, EMW < −61 ms having a sensitivity of 79% and specificity of 60% (AUC: 0.748). Adding QTc to EMW on the ROC analysis did not significantly affect the predictive accuracy (Table 3).

### 3.6 Treatment with B‐blockers

Between patients on and off B‐blockers, there were no statistical differences in QT, QTc, QAoC, and EMW intervals at rest (436 ± 47 vs 426 ± 43 ms; 452 ± 46 vs 454 ± 37 ms; 396 ± 45 vs 380 ± 47 ms and − 39 ± 22 vs −45 ± 23 ms, respectively, *P* > 0.2) or at recovery (450 ± 42 vs 463 ± 42 ms, 481 ± 38 vs 477 ± 33 ms; 395 ± 36 vs 387 ± 36 ms and − 58 ± 31 vs −75 ± 49 ms, respectively, *P* > 0.1 for all). At PE, patients on B‐blockers had less negative EMW (−66 ± 21 vs −113 ± 25 ms, *P* < 0.001), shorter QT interval (370 ± 27 vs 406 ± 25 ms, *P* < 0.001), and slightly longer QAoC (313 ± 23 vs 303 ± 4 ms, *P* = 0.05) than those on no treatment. QTc did not differ between the two groups (491 ± 37 vs 509 ± 52 ms, *P* = 0.1).

### 3.7 Genotype‐based analysis of response to exercise

LQTS genotyping is not always routine clinical practice, depending on patients' own preference or different management protocols. This is why initially we assessed the total LQTS group irrespective of the genotype. Whereby compare the two genotype groups (LQT1 and LQT2). There was no difference between LQT1 and LQT2 patients in QT, QTc, QAoC, and EMW values, neither at rest nor recovery (Table 4). However, LQT1 had longer PE QT and QTc than LQT2 patients with no differences noted in the QAoC interval (Table 4). As a result, PE EMW values were more negative in LQT1 (Table 4).

**Table 4:** Genotype analysis of response to exercise

Variable	LQT1 (*n* = 36)	LQT2 (*n* = 11)	*P*‐value
At rest			
QT rest	431 ± 44	433 ± 52	0.8
QTc rest	458 ± 15	451 ± 10	0.8
QAoC rest	390 ± 45	385 ± 54	0.7
EMW rest	−40 ± 23	−48 ± 18	0.3
At peak exercise			
QT peak	405 ± 25	381 ± 32	0.03
QTc peak	503 ± 47	458 ± 41	0.03
QAoC peak	308 ± 18	308 ± 19	0.9
EMW peak	−106 ± 25	−82 ± 34	0.04
At recovery			
QT recovery	455 ± 39	464 ± 51	0.5
QTc recovery	477 ± 34	488 ± 38	0.3
QAoC recovery	393 ± 34	389 ± 41	0.7
EMW recovery	−63 ± 45	−76 ± 35	0.4

Abbreviations: QTc, corrected QT interval by Bazett formula; QAoC, time interval from R onset to aortic valve closure; EMW, electromechanical window (QAoC‐QT); LQTS, long QT syndrome.

In LQT1 patients, QTc interval prolonged and EMW became more negative at PE in contrast to controls (Δ: +52 ± 45 vs −23 ± 16 ms, *P* < 0.0001) in whom EMW remained almost unchanged (Δ: −57 ± 19 vs −1.1 ± 8 ms, *P* < 0.0001). At recovery, QTc interval remained longer (ΔQTc: +23 ± 36 vs 1 ± 6 ms, *P* < 0.0001) and EMW remained more negative than baseline as opposed to controls (ΔEMW: − 22 ± 16 vs 7 ± 1 ms, *P* < 0.0001).

At PE, and in relation to baseline, QTc minimally prolonged in LQT2 patients in contrast to controls (ΔQTc: +2 ± 64 vs −23 ± 16 ms, *P* < 0.0001) and EMW became more negative (ΔEMW: −42 ± 37 vs −1.1 ± 8 ms, *P* < 0.0001). At recovery, QTc increased and EMW became more negative in LQT2 patients as opposed to controls (ΔQTc: +38 ± 9 vs 1 ± 6 ms, ΔEMW: −30 ± 12 vs 7 ± 1 ms, *P* < 0.0001).

ROC curve analysis comparing the total LQTS population with the LQT1 and LQT2 patients showed the following: (a) PE EMW may serve as a better predictor of cardiac events in LQT1 patients than in LQT2 or the population as a whole and (b) EMW at rest and recovery may better discriminate symptomatic LQT2 than LQT1 or the total LQTS population (Table 5 and Figure S5).

**Table 5:** ROC curve analysis of EMW performance in predicting previous cardiac events for the total LQTS population and for LQT1 and LQT2 patients separately

ROC analysis	Total LQTS	LQT1	LQT2	*P*‐value
EMW at rest				
AUC	0.757	0.748	0.900	*P* > 0.2
95% CI	0.615‐0.900	0.575‐0.877	0.576‐0.997	
EMW at peak exercise				
AUC	0.765	0.827	0.750	*P* > 0.3
95% CI	0.620‐0.910	0.664‐0.932	0.412‐0.950	
EMW at recovery				
AUC	0.748	0.743	0.867	*P* > 0.4
95% CI	0.603‐0.893	0.570‐0.874	0.536‐0.991	

Abbreviations: AUC, area under the curve, ROC, receiver operating characteristic; EMW, electromechanical window; LQTS, long QT syndrome; CI, confidence interval.

## 4 DISCUSSION

*Findings*: Our results show that in LQTS mutation carriers, there was reversed EM sequence with QT ending after aortic valve closure. As a result, EMW became negative at all three exercise phases, in contrast to controls where it remained positive. EMW also became more negative at PE and continued to be so during recovery. These abnormalities were more pronounced in patients with previous arrhythmic events and EMW was better associated with those events than QTc. B‐blockers seem to decrease the extent of EMW negativity during exercise mostly by shortening the QT interval but also by prolonging the QAoC duration. Analysis of our patients' according to genotypes showed LQT1 to have worse EMW negativity at PE than LQT2 patients, despite no differences at rest and recovery. To the best of our knowledge, this is the first study to use the EMW parameter to examine LV EM coupling response to dynamic exercise in genotype positive LQTS patients and to investigate the effect of B‐blocker therapy.

*Data interpretation*: Spatiotemporal EM heterogeneity is exaggerated in inherited LQTS^10^, ^27^, ^28^ with increased dispersion of myocardial repolarization during exercise and recovery preceding arrhythmias.^6^, ^20^ Mechanical heterogeneity, reflected by prolonged myocardial contraction and increased regional and transmural mechanical dispersion, is known to be more pronounced in symptomatic LQTS.^4^, ^15^, ^16^, ^29^ These EM coupling disturbances have been shown in the form of reversed (negative) EMW with mechanical systole ending before electric systole in symptomatic LQTS.^10^, ^30^ Our results confirm those findings in showing negative EMW at rest in LQTS, particularly in patients with previous arrhythmic events. We have also shown that those disturbances worsen with exercise as a result of a lesser degree of QT shortening over mechanical systole duration thus inadequate repolarization. Such behavior is in contrast to that in controls, in whom a parallel and analogous EM shortening occurs.^30^ As such, repolarization continues after completion of mechanical systole resulting in prolonged action potential duration and myocardial Ca^2+^ overload during diastole.^10^ These may generate early and late potentials, induce mechanical postsystolic contraction and predispose to tachyarrhythmias.^19^, ^20^, ^31^

The association of EMW negativity with arrhythmia has been shown in animal models of drug‐induced LQTS arrhythmia.^10^, ^19^, ^20^ EM coupling disturbances in the form of very negative (−200 ms) and dynamic EMW have also been reported after B‐adrenergic stimulation.^19^, ^20^ Ter Bekke et al showed that EMW may identify patients with previous arrhythmic events better than resting QTc.^17^ Our results indeed support those findings, not only with resting EMW having an accuracy of 0.757 vs 0.517, but also with respective values of 0.765 vs 0.569 at PE and 0.748 vs 0.407 during recovery. EMW particularly at PE had higher specificity in identifying high‐risk patients. The combination of QTc and EMW did not significantly affect the accuracy of the latter.

B‐blockers are widely used in the management of LQT1 and LQT2 patients due to their effect in decreasing sympathetic overstimulation on the myocardium and shortening the QTc.^32^, ^33^ Their use has been shown to be associated with reduced cardiac events.^32^ However, the risk remains in those who were symptomatic prior to treatment.^32^ In our study, B‐blockers appeared to have an objective benefit in making EMW less negative with exercise. Despite no differences in QT, QTc, QAoC, and EMW at rest or at recovery, patients on B‐blockers had significantly less negative EMW at PE, with no differences noted in QTc. Interestingly, this response was the result not only of QT shortening but also of QAoC prolongation. These results confirm previous experimental observations on drug‐induced LQT animal models.^19^, ^20^ Those studies along with our data reflect the potential preventive role of B blockers in patients with significantly deranged EM coupling.^17^ Thus EMW response to exercise may help identify LQTS patients who are at highest risk and may not respond to B‐blockers.

Finally, our results showed different patterns of EMW response to exercise between LQT1 and LQT2 patients, despite no difference in QT, QTc, QAoC and EMW at rest or recovery. EMW values at PE were more negative and QT and QTc intervals more prolonged in LQT1 patients. In the same group, QTc prolonged and EMW became more negative at PE and during recovery. However, changes in these parameters were not as pronounced at PE in the LQT2 subgroup. These findings may reflect the different response to adrenergic stimulation triggers between the two genotypes.^34^ In LQT2, genetic mutations are responsible for the malfunction of the rapidly activating component of the delayed rectifier potassium current (I_Kr_), which mainly controls repolarization at rest.^35^ However, in LQT1, defects in the slowly activating delayed rectifier potassium current (I_Ks_), affect the repolarization process during exercise.^10^, ^35^ The result is inadequate action potential shortening, manifested as prolonged QT interval, which combined with the mechanical effects (increased myocardial inotropy and lucinotropy) of adrenergic stimulation at PE, may explain the different EMW response between the two groups.^36^, ^37^

These findings may also explain the variations we noted in the predictive value of EMW in the three phases of exercise in the two genotype groups. Peak EMW was stronger for LQTI patients as opposed to the rest and recovery EMW in the LQT2 group. However, these results need to be seen with caution as the number of patients is small and cannot be generalized.

*Clinical implications*: Our study showed that EMW negativity at all three phases of exercise was more pronounced in the symptomatic LQTS patients. B‐blockers appeared to decrease the extent of EMW negativity at PE in LQTS. EMW is easy to assess and independently predicted previous arrhythmic events with higher sensitivity and specificity than QTc. Measuring EMW response to exercise increased the accuracy of stress echocardiography in identifying patients at risk of arrhythmias, thus may play a role in guiding towards optimum management.

*Study limitations*: Our study includes a modest number of patients and our results need to be reproduced in a larger cohort of patients with and without arrhythmic events and in relation to genotype. Limitations in defining the end of T wave may arise especially due to motion artifacts from exercise. Our proposed accuracy of EMW in predicting arrhythmia is based on the documented history we have in patients records rather than symptoms developing during exercise, except two patients in whom the exercise test has to be prematurely terminated due to signs of arrhythmia. PE heart rate was below the age predicted in controls and patients, with no significant difference between groups. While lack of fitness could be the explanation for low achieved heart rate in controls, it could also be the effect of B blockers which attenuated the heart rate rise in patients. The lack of difference between groups supports our potential explanations.

## 5 CONCLUSION

Cardiac EMW measurements correlate with QT interval, and reflect significantly reversed LV end systolic EM relationship in LQTS patients. These disturbances are worsened during exercise and early recovery and seem to be associated with previous arrhythmias. While EMW negativity is worse in symptomatic patients, it is less pronounced in those treated with B‐blockers. Thus, incorporating EMW assessment in the routine assessment of LQTS patients may help better stratification and symptom interpretation, even if only in some.

## CONFLICT OF INTEREST

No conflicts of interest.

## Supporting information


**Figure S1** Electromechanical window (EMW) calculation, EMW = QAoC–QT.

**Figure S2** Electromechanical window response to exercise for the two groups.

**Figure S3** Correlation between electromechanical window (EMW) and QTc.

**Figure S4** Electromechanical window response to exercise for the three groups (symptomatic long QT syndrome [LQTS], asymptomatic LQTS and controls).

**Figure S5** Receiver operating characteristic (ROC) curve analysis of electromechanical window (EMW) performance in predicting previous cardiac events.
