# Exercise worsening of electromechanical disturbances: A predictor of arrhythmia in long QT syndrome

**DOI:** 10.1002/clc.23216

**Published:** 2019-06-18

**Authors:** Dafni Charisopoulou, George Koulaouzidis, Annika Rydberg, Michael Y. Henein

**Affiliations:** ^1^ Institute of Public Health and Clinical Medicine Umea University Sweden; ^2^ Department of Paediatric Cardiology Leeds Teaching Hospitals NHS Trust Leeds UK; ^3^ Department of Cardiology Mid Yorkshire Hospitals NHS Trust Wakefield UK; ^4^ Department of Clinical Sciences, Paediatrics Umea University Umea Sweden; ^5^ Molecular and Clinical Sciences Research Institute St George University London London UK; ^6^ Brunel University Middlesex UK

1

*Clinical Cardiology*. 2019;42:235–240.

DOI: 10.1002/clc.23132

Michael Y. Henein's name was incorrectly listed in the article as Henein Y. Michael. The authors apologize for the error.

