# Supplementary figures and images for: Exercise worsening of electromechanical disturbances: A predictor of arrhythmia in long QT syndrome
Source: Clin Cardiol. 2018 Dec 22;42(2):235–40. doi: 10.1002/clc.23132 (PMC6712344; doi:10.1002/clc.23132)

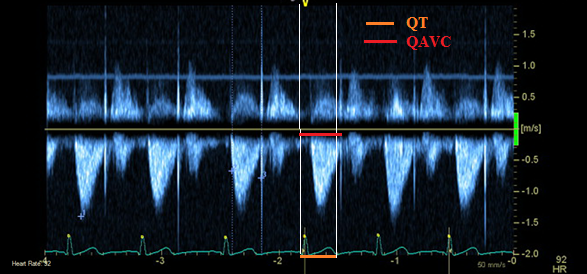

Supplement: Supplementary file 1 — Figure S1 Electromechanical window (EMW) calculation, EMW = QAoC–QT. [file CLC-42-235-s001.tif]

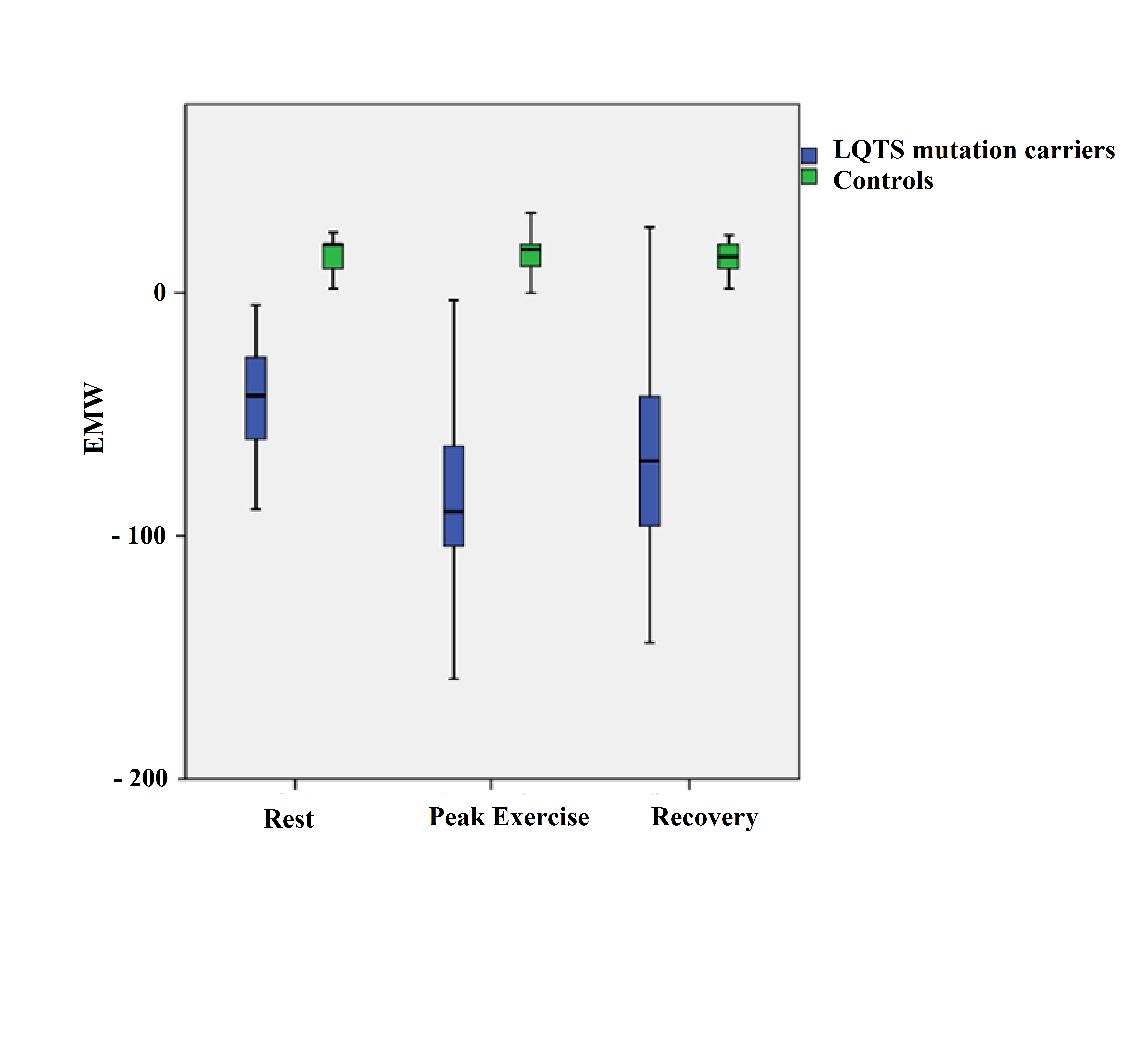

Supplement: Supplementary file 2 — Figure S2 Electromechanical window response to exercise for the two groups. [file CLC-42-235-s003.tif]

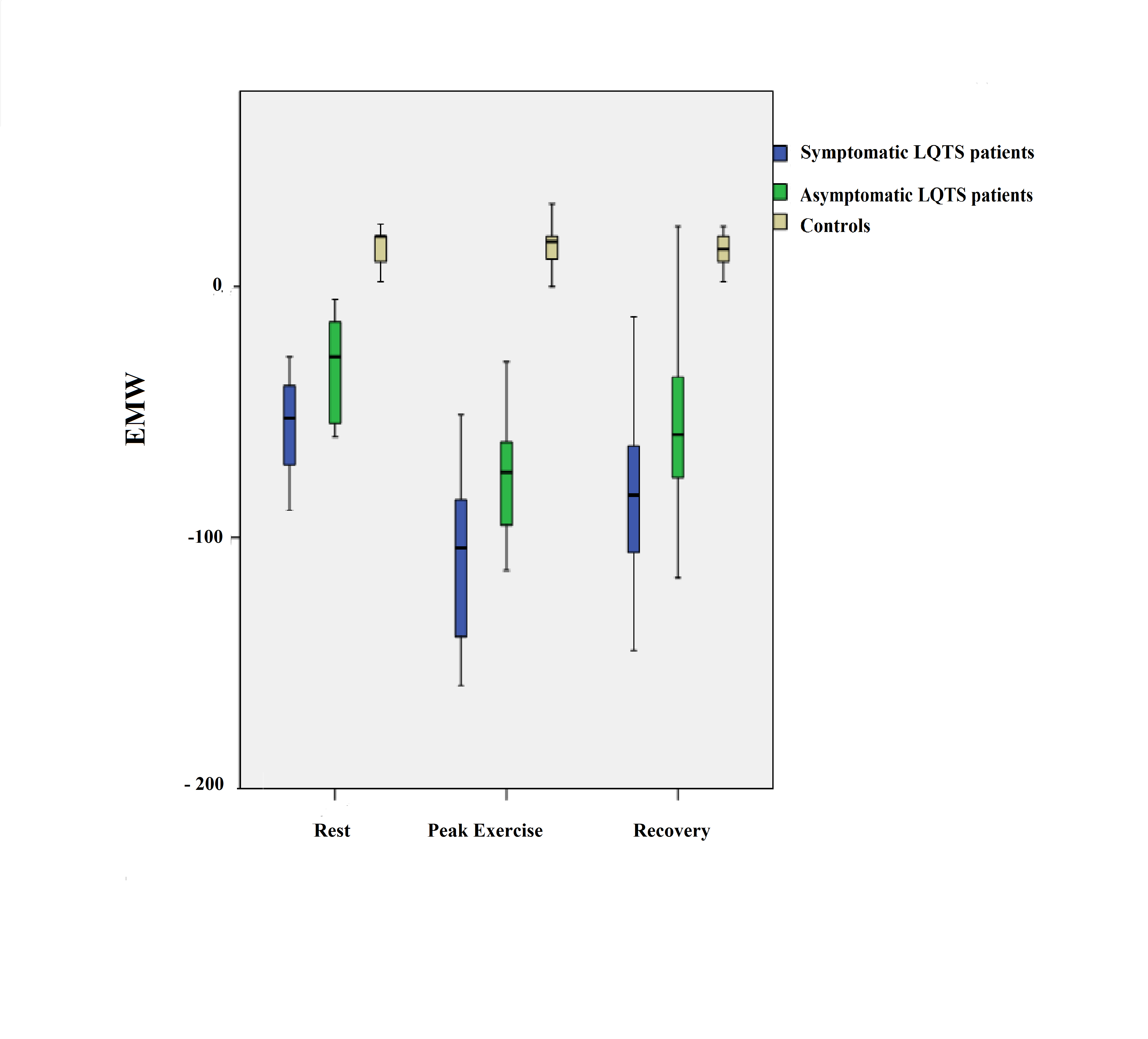

Supplement: Supplementary file 3 — Figure S3 Correlation between electromechanical window (EMW) and QTc. [file CLC-42-235-s002.tif]

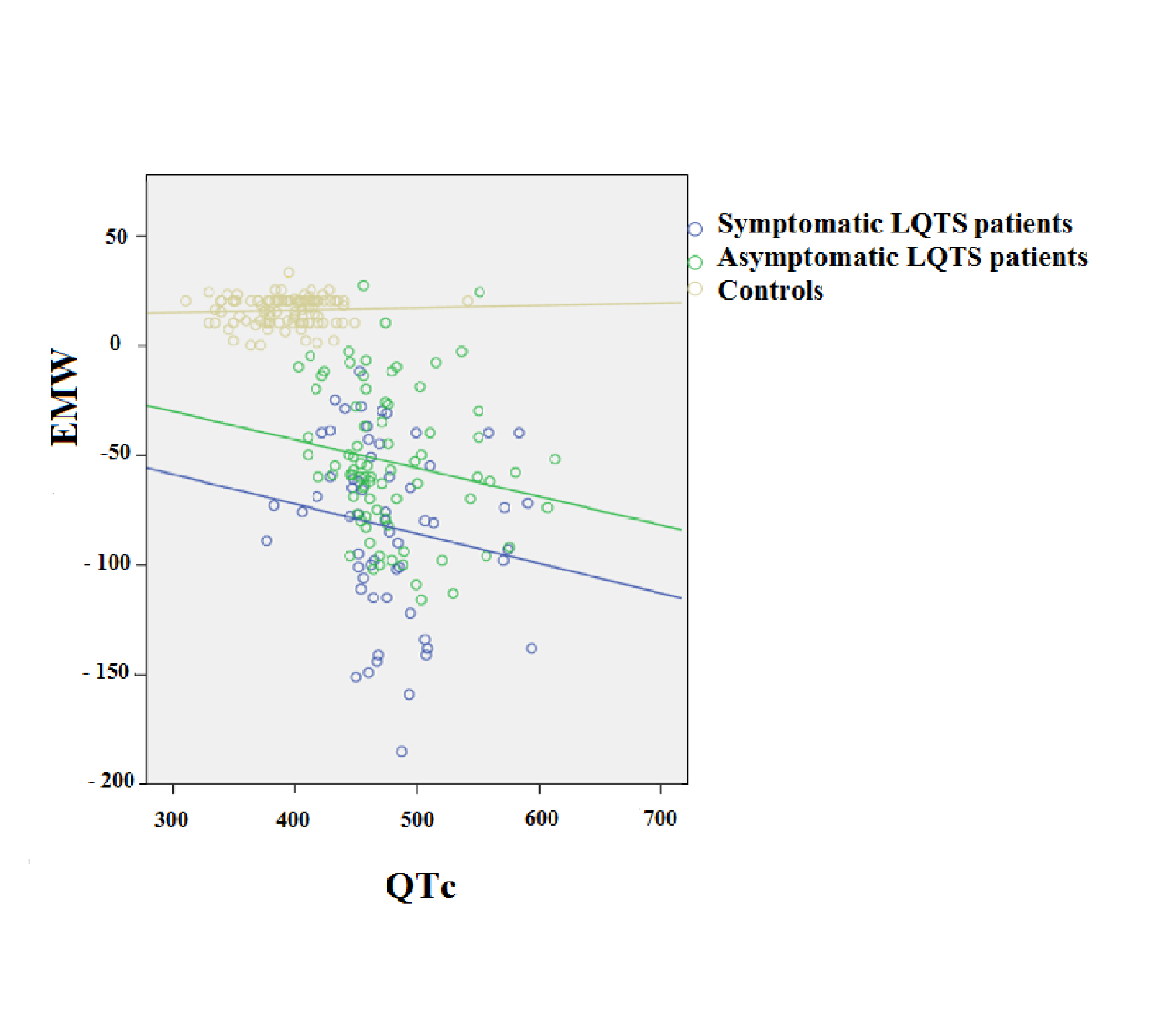

Supplement: Supplementary file 4 — Figure S4 Electromechanical window response to exercise for the three groups (symptomatic long QT syndrome [LQTS], asymptomatic LQTS and controls). [file CLC-42-235-s004.tif]

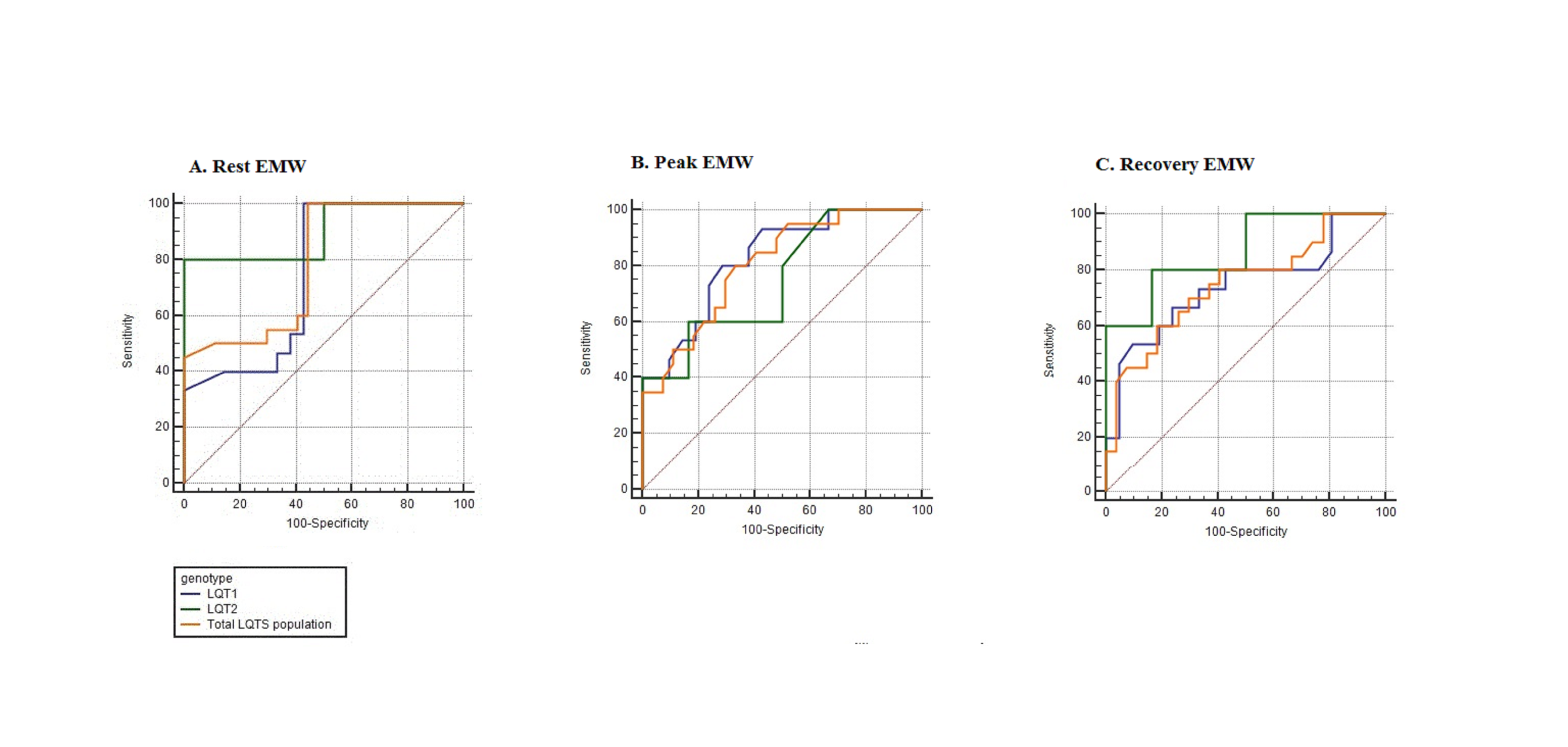

Supplement: Supplementary file 5 — Figure S5 Receiver operating characteristic (ROC) curve analysis of electromechanical window (EMW) performance in predicting previous cardiac events. [file CLC-42-235-s005.tif]
